# Quantifying and Visualizing Emergency Physician Workflow: Observational Time-Motion Study

**DOI:** 10.2196/85983

**Published:** 2026-07-22

**Authors:** Andrew J Henreid, Kimon L H Ioannides, Joshua M Pevnick, Tara N Cohen, Sam S Torbati, Teryl K Nuckols, Carl T Berdahl

**Affiliations:** 1 Department of Medicine, Cedars-Sinai Medical Center, 8700 Beverly Blvd, Los Angeles, CA, 90048, United States, 1 310-423-3141; 2 Department of Psychological Sciences, University of Connecticut, Storrs, CT, United States; 3 Department of Emergency Medicine, Cedars-Sinai Medical Center, Los Angeles, CA, United States; 4 Department of Emergency Medicine, University of California Los Angeles, Los Angeles, CA, United States; 5 Department of Surgery, Cedars-Sinai Medical Center, Los Angeles, CA, United States; 6 Division of General Internal Medicine, Cedars-Sinai Medical Center, Los Angeles, CA, United States

**Keywords:** emergency medicine, workflow, physicians, electronic health records, computers, patient care, documentation, efficiency, time management, time and motion studies

## Abstract

**Background:**

Emergency physicians face considerable workflow challenges in the emergency department (ED) due to unpredictable work environments, frequent interruptions, and mounting documentation requirements, all of which contribute to substantial computer-based work. Excessive time away from direct patient care is increasingly viewed as detrimental to the quality of care, communication, and patient safety.

**Objective:**

This study aimed to quantify and visualize how emergency physicians allocate their time during ED shifts, particularly the time spent on the computer.

**Methods:**

This cross-sectional observational time-motion study was conducted in a high-volume, urban ED. We used the validated TimeCaT application to continuously track physician activities, including computer use, direct patient care, communication, and all other tasks carried out during the shift. Electronic health record event log data were queried to estimate physicians’ computer use after their scheduled shift. The main outcome was the proportion of time spent on each activity. The primary measure was the total minutes of computer use (during and after the shift) per scheduled hour of clinical work. Additionally, we generated data visualizations to illustrate ED workflow-sequencing, task switching, and between-shift variability.

**Results:**

We observed 20 emergency physicians for one 8- to 9-hour clinical shift each, generating over 150.0 hours of real-time observation data quantifying physicians’ ED workflow. Physicians spent a median of 34.1% of their shift minutes using the computer (156.5, IQR 145.2-179.3 min) and 26.9% with patients (115.2, IQR 102.0-154.1 min). Other activities included 15.9% for verbal communication with staff (73.3, IQR 45.6-96.4 min), 9.5% phone use (41.4, IQR 33.4-59.2 min), 5.5% miscellaneous tasks (24.2, IQR 18.4-30.1 min), 3.9% personal time (16.8, IQR 9.9-25.4 min), 0.7% electrocardiogram review (3.8, IQR 1.4–5.0 min), and 0.4% other procedures (median 1.7, IQR 0.6-6.4 min). Electronic health record log analysis showed that physicians spent an additional median 1.3 (IQR 0.5‐2.6) hours using the computer after their scheduled shifts, for a combined 29.8 (IQR 25.6‐38.5) minutes on the computer per scheduled hour of their ED shift. Workflow visualizations showed frequent alternation among activity categories and noticeable variability in within-shift fragmentation across physicians.

**Conclusions:**

Emergency physicians spent more than one-third of their ED shift working on the computer, which was more time than they spent with patients. They also spent 1 to 2 hours using the computer after their scheduled shift. These findings demonstrate the need for strategies to reduce unnecessary computer use during and after clinical shifts to enhance workflow efficiency and improve patient care.

## Introduction

### Background

There is rising concern that spending too much time away from the bedside may undermine the quality of medical care by reducing opportunities for direct patient interaction and face-to-face communication among care team members [1-3]. Studies of emergency department (ED) physician workflow in the United States and internationally have reported that frequent interruptions, multitasking, and documentation demands are common in ED care and may contribute to cognitive burden, reduce efficiency, and raise important patient safety concerns [4-6]. The ED setting is among the most cognitively and operationally challenging work environments for physicians due to its high acuity, time pressures, and inherent unpredictability [7]. While prior literature has demonstrated that persistent interruptions and task switching can increase cognitive load and impair clinical efficiency [8-10], these aspects of workflow variability may also serve a functional purpose for timely information transfer and coordination of team-oriented emergency care [11].

In parallel, documentation and information management demands of modern emergency care have increased substantially with the widespread adoption of electronic health records (EHRs) [3,12]. Across various clinical settings, clinicians report spending large portions of their workday interacting with digital systems [12]. EHR work is not always finished within the time frame of a clinical shift, as many clinicians complete documentation and other computer-centered tasks after their scheduled shift, which may further contribute to workload and burnout [13]. Quantifying both on-shift and after-shift EHR work is therefore critical for evaluating EHR use, understanding workflow burden, and informing the design of interventions to improve efficiency and quality while supporting care team coordination.

A foundational step for improving ED work systems is to measure and describe how clinicians allocate time and sequence workflow activities during their clinical shifts. The application of methods from human factors engineering, such as time-motion studies, can help identify targets for workflow improvement and optimize work process design [14-16]. However, because direct observation alone does not account for after-hours work and EHR tracking cannot precisely capture noncomputer activities, an integrated approach that combines structured time-motion observation with EHR use data leverages the strengths of both modalities to more comprehensively characterize ED physician workflow and establish baseline measures for evaluating future workflow interventions.

This study provides a detailed assessment of ED workflow for emergency physicians, focusing on the balance of time spent at the computer versus the patient bedside. Using an integrated measurement approach that combines structured time-motion observation with EHR event log data, this work offers a reproducible mechanism to inform workflow evaluation, EHR design, and interventions aimed at reducing documentation burden while facilitating effective care delivery. Prior ED time-motion research has often concentrated on specific activity subsets or task patterns, such as medication-focused workflows [17]. This investigation, unlike previous ED time-motion studies that quantify only observed on-shift activity, (1) pairs structured time-motion observation with EHR event logs in the same physician cohort to capture both on-shift and after-shift work, (2) uses a validated structured observation tool with a task taxonomy for standardized measurement, and (3) provides workflow visualizations that illustrate how within-shift task switching and workflow heterogeneity occur across physicians.

### Objectives

The objective of this descriptive observational time-motion study was to assess and characterize emergency physician workflow by quantifying the time ED physicians allocate to clinical and nonclinical tasks. This was accomplished by (1) measuring the proportion of physicians’ time allocated to major on-shift activities using structured time-motion observation and (2) quantifying after-shift work using EHR event logs to track computer-based activity. We created data visualizations of on-shift workflow sequences to illustrate the variability in task density and fragmentation across physicians and their ED shifts in an effort to inform future work system design.

## Methods

### Study Design and Setting

We conducted a cross-sectional observational time-motion study and reviewed EHR event logs to track emergency physician activities in an urban, high-volume ED that is a comprehensive stroke center, ST-elevation myocardial infarction receiving center, and level-1 trauma center with an annual census of 90,000 ED visits. Daytime shift observations were conducted between May 21, 2019, and November 6, 2019. During the study period, attending emergency physicians supervised or provided care for all patients. Residents from nonemergency medicine specialties (internal medicine, anesthesia, and so on) assisted with care for less than 5% of all visits, and physician assistants were involved in 20%. The number of emergency physicians staffing the ED varied from a minimum of 3 during overnight shifts to a maximum of 6 during peak hours.

### Recruitment

Physicians were recruited through convenience sampling and provided consent to participate in the study in a private setting prior to their observed shift [18]. Participant eligibility criteria included emergency physicians credentialed to practice in the institution’s ED. No additional exclusion criteria were applied to this study sample.

### Ethical Considerations

This study, involving human participants, was approved by the institutional review board (IRB) of Cedars-Sinai Medical Center (Pro00057066) prior to data collection. The IRB reviewed all recruitment and consent processes prior to recruitment; no concerns were identified beyond standard privacy and confidentiality safeguards. Written informed consent was obtained from each participating physician before their observed shift [18]. The IRB approved a waiver of consent for patients, as the primary focus of the study was to observe physician activity where direct contact with patients and sensitive information was expected to occur only incidentally. All collected data were stored on secure institutional servers. Participants did not receive compensation for this study.

### Measures

The main outcome was the proportion of shift time spent in each activity category. For each observed shift, we calculated the proportion of observed shift time spent in each prespecified activity category recorded in TimeCaT. The primary measure of interest for this investigation was the total minutes of computer time per scheduled hour of the physician’s clinical shift. We calculated total computer time per scheduled hour as the sum of (1) directly observed on-shift computer time and (2) after-shift active EHR time from the event logs, divided by scheduled shift hours. The calculation of total computer time included the number of minutes each physician spent on the computer, both during and immediately after their scheduled ED shift. Activity categories included the percentage of time spent on specific shift activities. These categories consisted of computer usage (computer), direct patient interaction (patient), verbal communication with clinical staff (verbal), operating the phone (phone), miscellaneous tasks (miscellaneous), personal tasks (personal), EKG review (EKG), and performing clinical procedures (procedures).

EHR event log data provided detailed time stamps for all user actions carried out within the EHR computer system (eg, “ED Disposition Activity viewed,” “User logged in,” and “Notes viewed”) after each observed shift. EHR use was defined as the number of seconds each physician spent actively using the EHR during their event log timespan, using a 1-minute threshold of inactivity [19]. The physician’s EHR use was thus considered inactive if more than 59 seconds elapsed between any 2 successive EHR actions, indicating the physician had left the workstation or diverted their attention to perform other tasks [19,20]. Total active use was determined by calculating the time intervals between access times (access instants) for every active EHR action (ie, the number of seconds between every 2 subsequent logged events).

**Figure 1.**
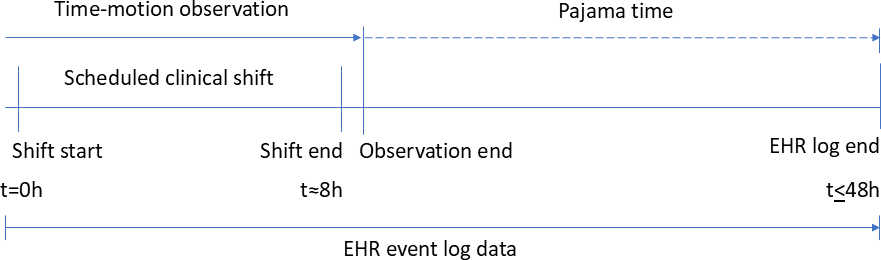
Overview of data collection process. Figure depicts the data collection timeline. In-person time-motion observation began at shift start (t=0) and continued until observation end. After-shift EHR activity (“pajama time”) was captured from observation end until up to 48 hours later or the start of the physician’s next clinical shift, whichever came first. Pajama time reflects active EHR use after the shift observation period ended, where t represents time markers in the combined data collection period (ranging from 0 h when the observed shift began to 48 h after the observed shift). EHR: electronic health record.

Pajama time is a term attributed in academic literature to characterize physician time spent using the EHR (computer) after the end of their scheduled shift (ie, on administrative actions such as writing clinical notes) [13,21,22]. In this study, we defined pajama time as any active EHR use that occurred after the observed shift concluded, captured up to 48 hours from the end of observation or the physician’s next clinical shift [13,21,23]. Pajama time was measured using EHR event logs capturing EHR actions up to 48 hours after the observed shift day (2-d span), which included EHR use occurring either on-site at the medical center or elsewhere (ie, at home). See Figure 1 for a depiction of the combined data collection methods used in this study*.*

### Data Collection

#### Time-Motion Observation Data

In-person, time-motion observations were conducted to collect workflow data characterizing the time ED physicians spent on prespecified tasks during ED shifts (one observed shift per enrolled physician). A single, trained observer with experience in research and clinical care as an emergency medical technician shadowed enrolled emergency physicians during clinical shifts scheduled to last 8 or 9 hours. Prior to data collection, the observer completed hands-on training to apply the ED task taxonomy and operate the data capture application, including a pilot session to practice real-time shadowing, task classification, and recording task transitions in the ED. Any ambiguities in task categorization and multitasking decision rules were immediately recorded using the application’s native time-logged note-taking and annotation features and were discussed with the research team. An observation protocol, operational definitions, and decision rules were established before official data collection and were followed throughout the study to ensure consistent application of standardized task definitions across shifts.

The observer began data collection at the time the shift began and ended only when the physician informed the observer that they were done interacting with patients and planned to either leave the ED or use the computer without further patient interaction. The observer took a 45-minute break from data collection approximately halfway into each shift, and the length of the shift was adjusted to account for this period prior to analysis.

**Figure 2.**
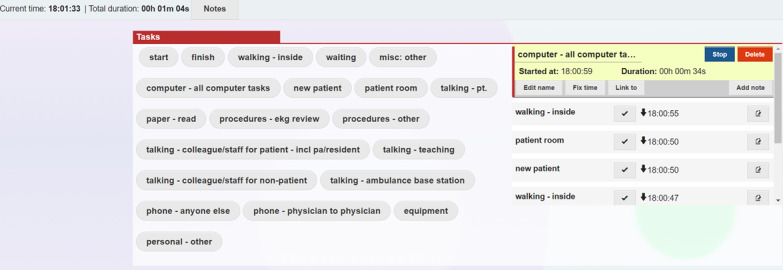
TimeCaT data capture interface (Time-motion). The figure presents an example of the TimeCaT 3.9 interface used for in-person observation data collection (time-motion) from the observer’s perspective.

The trained observer used a digital tablet and a web-based time-motion application (TimeCaT 3.9) to track start and end times (to the nearest second) across a list of prespecified tasks [24]. Time-motion studies are a well-established methodology for workflow research and are considered “gold standard methods” for health care observations [14]. Similarly, the TimeCaT application used for in-person data collection was specifically designed and validated for measuring workflow in health care contexts [24]. See Figure 2 for the graphical user interface used for in-person observation data collection.

Physician workflow (defined as the series of activities carried out by the physician during their shift) [25] was continuously assigned to one dominant task by clicking an activity-specific button in the TimeCaT application. These tasks included computer use, direct patient interaction, variations of phone use, EKG review, other clinical procedures, verbal communication with hospital staff, verbal instruction to staff, reading or writing on paper, waiting for the next task, gathering equipment, walking within the medical center, personal time, and miscellaneous actions. For clarity and comprehension, individual tasks were then combined into the following activity categories: computer use (computer), patient interaction (patient), verbal communication with ED staff (verbal), operating the phone (phone), EKG review (EKG), clinical procedures (procedures), all personal tasks (personal), and any remaining miscellaneous tasks (miscellaneous). See Table 1 for details on how individual tasks were classified into major activities.

**Table 1.** Categorization of physician activities (time-motion).

Activity category	Individual task
Computer	Computer—all computer tasks
EKG^a^	Procedures—EKG review
Miscellaneous	Miscellaneous: other Paper—read Waiting Walking—inside
Patient	New patient (unused) Patient room Talking—patient
Personal	Equipment Personal—other
Phone	Phone—physician to physician Phone—anyone else
Procedures	Procedures—other (ie, non-EKG)
Verbal	Talking—colleague or staff for patient Talking—teaching Talking—colleague or staff for nonpatient Talking—ambulance base station
Excluded research admin tasks	Start Finish

a EKG: electrocardiogram.

When the physician entered patient rooms, the observer waited outside the room in the interest of patient privacy, and the time spent inside the patient’s room was assumed to be spent on direct patient care. TimeCaT did not include an explicit multitasking measurement in our study’s configuration but captured it indirectly as a rapid set of alternations between tasks. When physicians were performing multiple tasks simultaneously (eg, talking on the phone while using the computer), the observer determined which task was the dominant focus of the physician’s attention and alternated accordingly. This, in addition to the highly variable nature of ED workflow, resulted in many transitions between tasks during periods of multitasking, which the TimeCaT software was designed to capture [24].

#### Event Log Data From the EHR

To supplement the time-motion data collected during in-person observations, we also obtained EHR event log data to estimate the amount of time physicians spent on the EHR after the end of each physician’s observed shift [12,20,26]. Each event log contained a detailed list of actions performed within the EHR by a particular user over a specified period of time, and each action was accompanied by a time stamp [12,26-29]. We collected the EHR event log data from the time in-person observation ended until up to 48 hours later or the time that the physician’s next shift began (whichever was earlier). One physician’s EHR event log data was excluded from the “pajama time” analysis because their unique administrative responsibilities suggested that their EHR actions were not directly related to their clinical care.

### Analysis

Descriptive analyses were performed using Microsoft Excel and SPSS Statistics (version 24.0; IBM Corp). For each observed shift, we calculated the total time spent in each activity category from TimeCaT’s time-stamped task interval data export. Per-shift activity proportions were computed as the time spent in a given category divided by the total observed shift time. We summarized shift-level proportions across physicians using medians and IQRs. For the primary measure of computer use (computer time per scheduled hour), we calculated observed on-shift computer time plus after-shift active EHR time, divided by scheduled shift hours. After-shift active EHR time was computed from audit event logs using a 1-minute inactivity threshold between logged events, as described in the “Measures” section.

Data visualizations were created using Tableau Desktop, Microsoft Office, and Seaborn [30]. The data visualizations were used to illustrate physician workflow allocation by activity type and the sequence of activities over the course of each ED shift.

## Results

### Overview

Twenty attending emergency physicians were enrolled in the study (5 female and 15 male). The median number of years in practice since residency graduation was 14.5 (IQR 7.5‐23.0; range 3-44 y), with a residency graduation year range of 1976-2017. We collected a total of 150.0 hours of time-motion data, with a median observation duration of 7.3 (IQR 7.2‐8.1) hours per physician. This included a total of fourteen 8-hour shifts and six 9-hour shifts, with a shift start time range of 5 AM to 1 PM.

EHR event log data were obtained for all 20 clinical shifts, starting from the time the shift observation ended and continuing for up to 48 hours or until the beginning of the physician’s next clinical shift. Initially, our dataset of EHR event logs included descriptions of physician actions over a total of 200.3 hours including pajama time. After the exclusion of 1 physician with unique administrative responsibilities, EHR event log data used in the reported analysis included a total of 189.1 hours (median 10.1, IQR 8.5‐10.6 h per physician) across the remaining 19 physicians in the sample.

### Outcomes

For the primary outcome measure of total computer time per scheduled hour of clinical shift, we found that ED physicians spent a median of 29.8 (IQR 25.6‐38.5) minutes on the computer per hour of scheduled clinical work. Total minutes spent on the computer included both directly observed computer time during the ED shift and EHR pajama time that occurred after the physician’s observed shift.

#### Direct Observation of On-Shift Activities

Based on real-time observational data across all 20 ED shifts, emergency physicians spent a median of 34.1% of their shift on the computer (156.5, IQR 145.2‐179.3 min). The longest interval of time away from the computer at any point during a shift was 20.0 minutes (range 8.0-20.0). Secondary to computer use, physicians spent a median of 26.9% of their shift with patients (115.2, IQR 102.0‐154.1 min). Among all 20 physicians, the longest time continuously spent at a patient’s bedside for any physician was 21 minutes 47 seconds (range 7.4-21.8 min).

Additionally, ED physicians spent a median of 15.9% of their shift verbally communicating with staff (73.3, IQR 45.6‐96.4 min). Physicians spent a median of 9.5% of their total shift using the phone to talk to other health care workers (41.4, IQR 33.4‐59.2 min), with a median duration of 1.1 minutes per phone task. Miscellaneous clinical tasks constituted a median of 5.5% of their shift (24.2, IQR 18.4‐30.1 min). Clinical procedures included 0.7% EKG review (3.8, IQR 1.4‐5.0 min) and 0.4% for other procedures (1.7, IQR 0.6‐6.4 min). Time spent on EKG review is described in more detail in a related manuscript that focuses on interruptions [31]. Across all physicians, personal time taken while working constituted only 3.9% of their total shift workflow (median 16.8, IQR 9.9‐25.4 min) and included time spent eating, using the restroom, and personal smartphone use.

**Figure 3.**
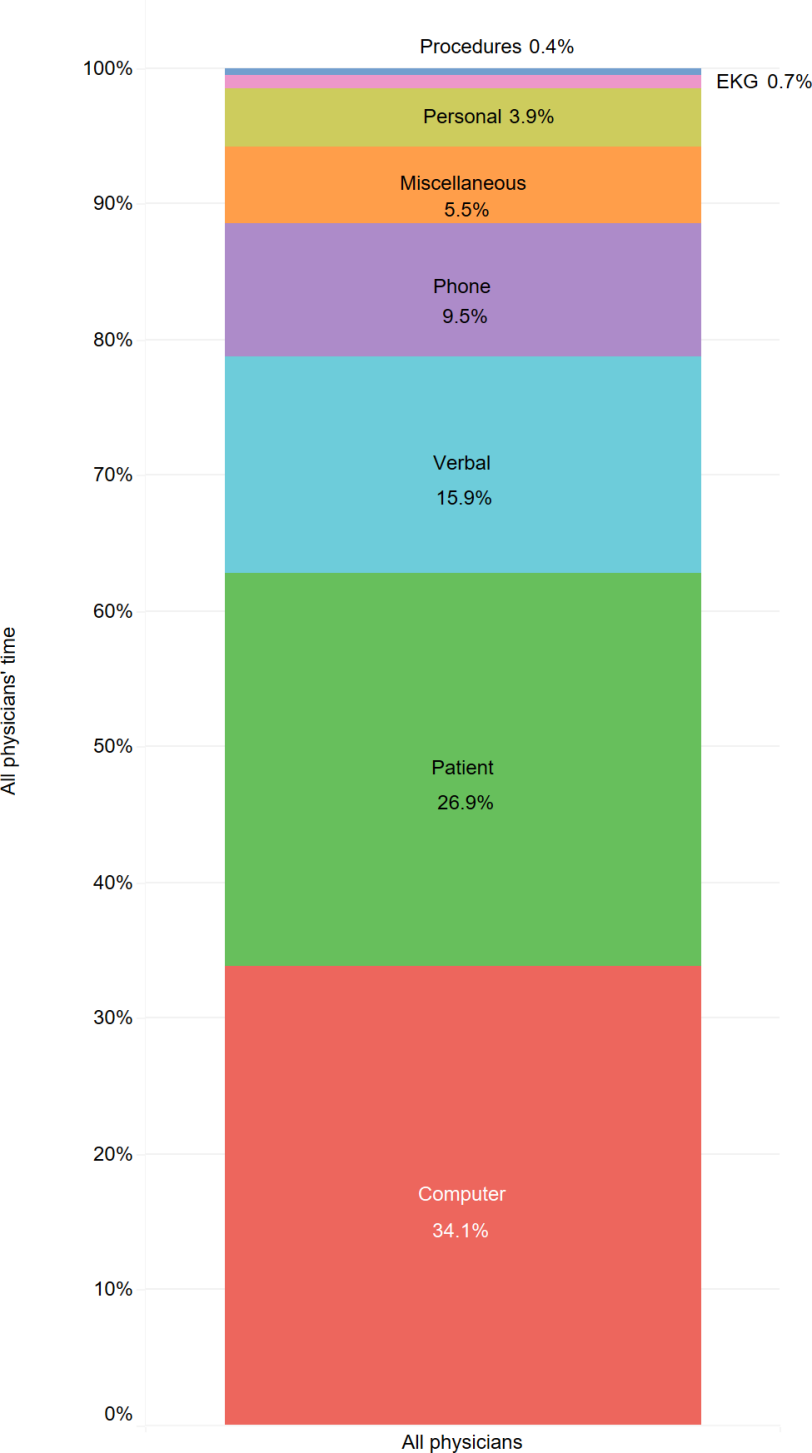
Allocation of physician time in the ED (Percentage of total shift across 20 observed physicians). The visualization illustrates the cumulative allocation of physicians’ time on shift by activity category and across all observed physicians. ED: emergency department; EKG: electrocardiogram.

**Figure 4.**
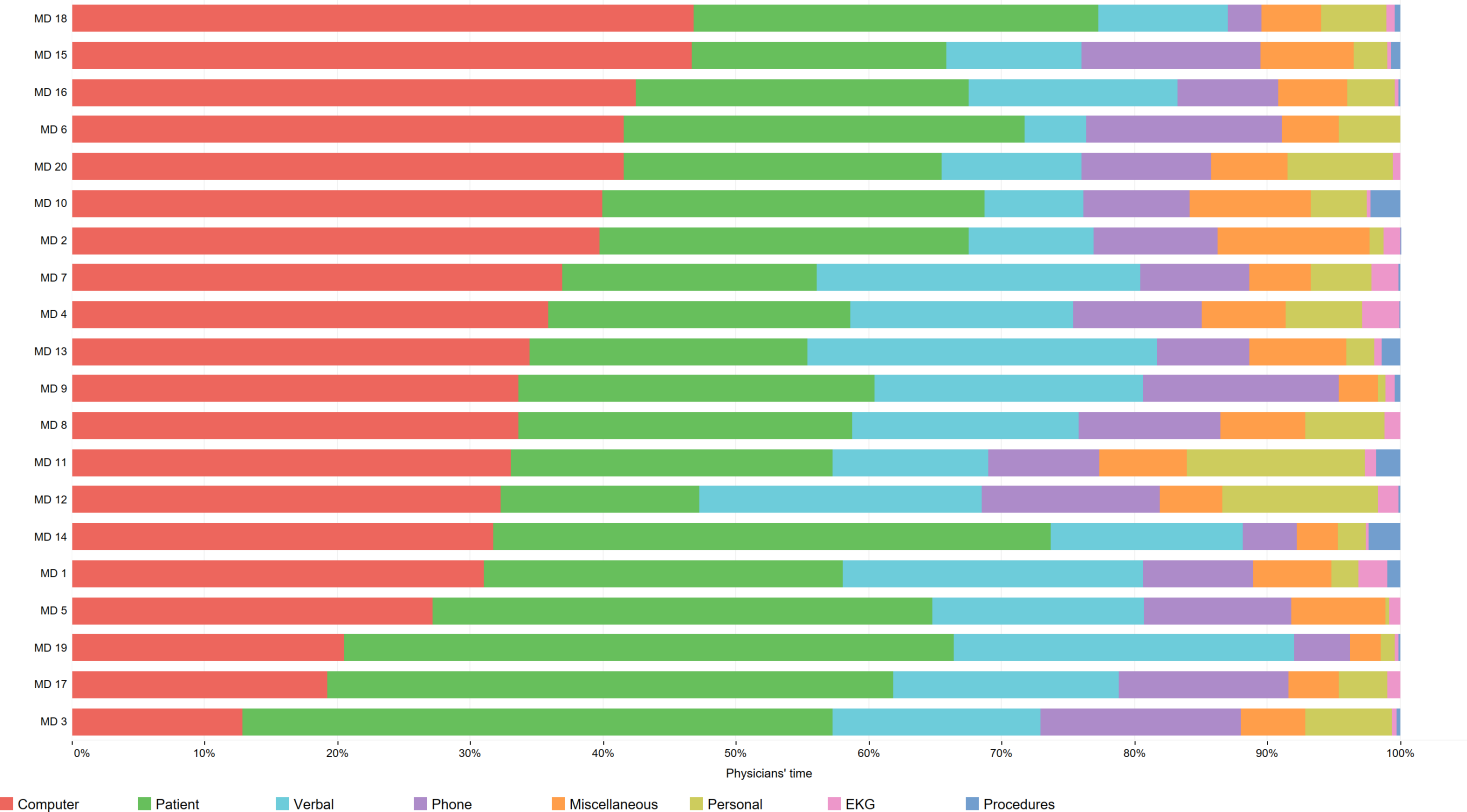
Allocation of physician time in the ED (By physician). Visualization illustrates the individual allocation of physicians’ time on shift by activity category and observed physician. Individual stacked bars are sorted in descending order by the percentage of computer use. ED: emergency department; EKG: electrocardiogram.

**Figure 5.**
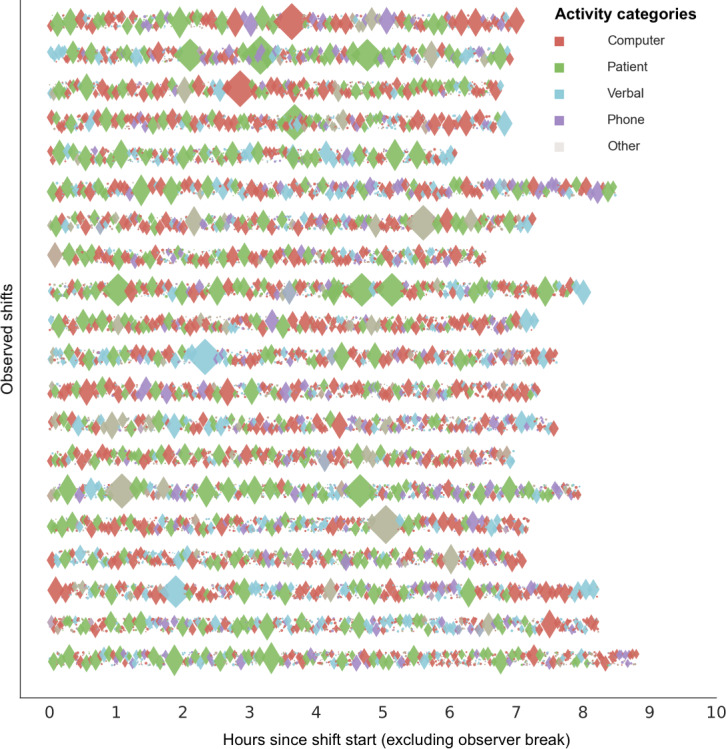
Timelines of physician workflow in the ED (By physician). The visualization illustrates the timing, sequence, and duration of observed activities during each observed ED shift (N=20). The x-axis shows the time since the shift start (time=0 bottom left) in hours of observed time. Each horizontal row corresponds to one observed physician shift. Shifts are ordered from top to bottom by the total number of recorded tasks (top row=fewest tasks; bottom row=most tasks). Each diamond represents a single observed task; diamonds are plotted at the task start time, color-coded by activity category (legend top right, with the 4 least common categories merged into a single gray color for visual clarity), and scaled in size to represent task duration (larger diamonds=longer tasks). Diamonds are displayed with a slight vertical “jitter” within each row to reduce overlap between closely timed tasks and enhance visual clarity. This visualization is intended to provide a task-level view of workflow fragmentation and task switching over time, and to illustrate variability in workflow sequencing across observed shifts. See Multimedia Appendix 1 for timelines depicting all task category instances. ED: emergency department.

Data visualizations of physician workflow illustrate the substantial allocation of time spent on the computer compared to other clinical priorities, as well as variations in workflow between and throughout ED shifts. See Figures 3-5 f*o*r visualizations of physicians’ time by activity (Figure 3), by individual shift (Figure 4), and linearly across each shift (Figure 5). The resulting Figure 5 (timelines of physician workflow in the ED) provides a task-level visualization of observed shift activities over time. Each row of the figure represents one observed ED shift, with each diamond indicating a task instance plotted at its measured start time, the color denoting the activity type, and the diamond size reflecting task duration. To improve visual clarity, the least prevalent task categories are depicted in gray (timelines depicting all task instances can be found in Multimedia Appendix 1). This depiction shows frequent alternation among activity categories (task switching) and substantial variability in fragmentation between ED shifts, with some shifts characterized by many high-intensity activity intervals and others containing longer, uninterrupted task periods.

#### After-Shift Computer Use

Across 19 clinical shifts, we obtained data on 112,396 EHR log events (actions) with a median of 5732 (IQR 5213‐6519) events per physician and an overallevent rate of 9.9 events per minute. The total EHR log timespan recorded from this data, including active and idle computer use, was approximately 189.1 hours across 19 included physicians. After applying the 1-minute inactivity threshold to calculate total active EHR use after the shift, physicians spent a median of 1.3 (IQR 0.5‐2.6; range 0.0-5.3) hours on pajama time after the end of their scheduled shift.

Across all logged EHR events, we identified 147 unique EHR actions from the metric names and descriptions present in the audit log, with a median of 57.5 (IQR 50‐62) unique EHR actions per physician. The most frequently logged EHR actions included: “Report with patient data viewed,” “SmartLink used,” “Visit Navigator template loaded,” “Inpatient system list accessed,” and “ED Disposition Activity viewed.”

## Discussion

### Principal Results

In this descriptive observational time-motion study of emergency physician workflow in a high-volume, metropolitan, level-1 trauma center, our results reveal that physicians spent more time using computers than engaging in direct patient care—generally spending more than a third of their shift on the computer or approximately half of every scheduled hour when accounting for “pajama time” after their shift. Our results demonstrate that the baseline work system in our ED is complex, involves frequent task switching, and includes substantial time at computer systems. The workflow visualizations presented can be used to support interpretation and guide discussions around ED workflow, including how organizational or health IT changes might influence these patterns. Figure 5 and Multimedia Appendix 1, in particular, provide a task-level depiction of activity sequencing, which can be used as a visual aid to help communicate workflow complexity to clinicians, administrators, and nonclinical stakeholders. These findings add to a growing body of evidence that time spent on direct patient care is limited by competing workflow demands (especially the burden of computer use) associated with ED care delivery [32-36].

These findings identify concrete targets with informatics implications for workflow and EHR improvement in emergency care. The high proportion of computer-based work suggests a need to reduce low-yield documentation and user burden through usability-focused redesign, such as minimizing unnecessary EHR flows for common tasks while preserving team coordination. The task-sequence visualizations also highlight substantial within-shift fragmentation and heterogeneity in task density across shifts, suggesting that interventions should be evaluated not only on total computer time but also on whether they reduce excessive task switching. Finally, because this study integrates structured time-motion observation with EHR event log data in the same cohort, it provides a reproducible measurement approach that can be used to evaluate EHR usability and workflow interventions using consistent, prespecified outcomes.

Direct observation and audit logs have complementary strengths and limitations; combining them provides a more complete characterization of ED workflow than either method alone. Our study combines data from both in-person time-motion observations and EHR event log data to demonstrate that the burden of computer use is not limited to time on-shift but also commonly extends hours beyond the end of clinical shifts [12,13,22]. While EHRs facilitate improved patient care by providing physicians access to patients’ prior medical histories and test results, for example, they also complicate the clinical work environment and may lead to unintended consequences such as limiting physician time at patients’ bedsides [35,37].

We undertook this study to establish baseline measurements describing the distribution of time spent at the computer and across other activities to prepare for the introduction of policy changes and technologies that could alter workflows. During the study period, our emergency physicians used voice recognition software to assist with producing clinical documentation, including written medical histories, physical examinations, interpretations of diagnostic test results, and medical decision-making notes. While some US documentation requirements have recently been revised to make them less burdensome, emergency physicians are still incentivized to write potentially lengthy medical decision-making notes that fully characterize the complexity of patient encounters so that their institutions can recover the maximum amounts available for the care of each patient [38,39].

Currently available EHR systems were designed to reflect what was previously possible using handwritten notes in paper filing systems, enable the collection of information necessary for billing, and facilitate improved access to essential clinical information. Possibly as a result of this history, persisting user interface and design limitations may place an additional cognitive burden on emergency physicians [40-42]. Usability improvements that are particularly relevant in the ED include reducing clicks and screen changes for common workflows (eg, reviewing results, documenting encounters, and placing orders), facilitating efficient retrieval of time-sensitive data, and minimizing redundant documentation. Streamlining team communication and task coordination within the EHR may also reduce fragmentation without compromising clinical coordination. Overly complicated interfaces and unintuitive action sequences in EHRs may frustrate physicians and increase the risk of medical errors [43-45]. Pragmatic, user-centered design principles should be incorporated as EHR systems are designed and tested, especially for those employed in ED care settings where time-critical actions are commonplace [35].

The development of more user-friendly interfaces that support seamless compliance and mitigate risk could significantly alleviate existing administrative pressures and allow physicians to focus more on patient-centered care [44,46]. Developing these systems requires additional research to understand how clinicians’ varied current and future approaches to EHR use may impact the quality of care, for example by assessing whether more “staccato” (frequently interrupted) or, conversely, “legato” (fewer, longer periods of use) interactions are associated with increased efficiency or errors. There is an urgent need for improved EHR design, documentation processes, and other human factors-centered solutions that enhance efficiency and improve quality and safety for patients [35,47,48]. Investing in “elbow-to-elbow” EHR training for emergency physicians may improve understanding of EHR functionalities, thereby enhancing their efficiency and reducing the associated burden for current and future clinicians [49-51].

Emerging technologies designed to decrease documentation burdens have shown early promise, although with some potential downsides. For example, ambient artificial intelligence (AI) scribes use natural language processing to transform real-time audio-recordings of clinical encounters into clinical documentation [52], and this technology is likely to advance rapidly due to advancements in AI capabilities over the last several years [35]. A recent study conducted at sites in 6 different health systems demonstrated an association between the introduction of ambient AI scribes and a reduction in pajama time [53]. However, careful evaluation of such products must be undertaken to understand the strengths and limitations of the technology and identify unintended consequences for physicians (ie, novel unexpected workflow challenges) as well as patients [54]. Authors of a noteworthy study conducted by Kaiser Permanente reported that early tests of ambient AI scribes were not well-integrated into their EHR at the time of testing, which led to technical difficulties and frustrations due to wasted time [52]. Regardless of specific technological enhancements, proposed workflow solutions should be evaluated against the outcomes used here (on-shift activity distribution, total computer time, and after-shift EHR time) to ensure improvements accurately reflect efficiency rather than redistribution of burden.

Overall, our study underscores the importance of undertaking more rigorous future human factors studies to improve workflow measurement in complex work environments as new technology is introduced, systematically assessing benefits and addressing drawbacks for clinical work. The Cognitive Work Analysis (CWA) framework was recently described as a method to “model and design sustainable sociotechnical ED systems” as new technologies are introduced [55]. Taken together with the Dynamic Sustainability framework [56], administrators and early adopters of technology should anticipate the need to conduct ongoing assessments of how sociotechnical systems are changing so that the needs of clinicians and patients are uncovered and addressed as technologies and tasks evolve over time.

### Limitations

Our study has several limitations. First, this was a single-site study, which may limit the generalizability of our results. Additionally, observations were conducted by a single trained observer; however, we used standardized task definitions and a structured, validated observation tool designed to handle task classification discrepancies [24]. Future studies could incorporate multiple observers and formal reliability testing when resources permit. Second, we recruited a convenience sample of attending physicians working day shifts to participate in the study. As such, it is possible that physicians who opted to participate have systematic differences in the way they spend their time compared to those who did not participate, and the findings may not generalize to overnight ED workflow. Physicians in this study varied in years of experience, which may be reflected in their workflow patterns. While our sample was not designed for statistical adjustment, future studies should investigate experience-related differences and night shift variability in workflow. Third, because our observer was instructed not to enter patients’ rooms due to privacy concerns, we assumed that time in rooms was entirely spent on direct patient care. Thus, it is possible that we overestimated the amount of direct patient care and underestimated the amount of time spent on other activities such as computer use and phone calls that could have occurred within patient rooms. Fourth, to incorporate EHR event log data into our analyses, we selected inactivity cutoff times based on qualitative comparisons of EHR event log data and in-person observation data that nonetheless may systematically overestimate or underestimate EHR time; however, similar inactivity thresholds have been used for EHR event data in existing literature [19,20]. Lastly, some physicians may have been using pajama time to catch up on documentation from prior shifts, which could have led to overestimation of total computer time. As we could not directly observe physicians after they left the ED, the use of EHR event logs with validated activity thresholds was found to be the most suitable approach for estimating postshift EHR use that would have otherwise gone unmeasured [19,20].

### Conclusions

This time-motion study found that emergency physicians allocate more than one-third of their time to computer use during their ED shift. We analyzed EHR event logs to estimate that physicians also spend an additional 1 to 2 hours using the computer after their scheduled shift. In total, emergency physicians spend approximately half of every scheduled shift hour on computer-based work. Future research should investigate strategies to minimize low-yield time at the computer and increase time spent providing direct patient care.

## Supplementary material

10.2196/85983 Multimedia Appendix 1 All-Task Timelines of Physician Workflow in the ED (By Physician): Visualization illustrates timing, sequence, and duration of observed activities during each observed ED shift (n=20). The x-axis shows time since shift start (time=0 bottom left) in hours of observed time. Each horizontal row corresponds to one observed physician shift. Shifts are ordered from top to bottom by the total number of recorded tasks (top row = fewest tasks; bottom row = most tasks). Each diamond represents a single observed task; diamonds are plotted at the task start time, color-coded by activity category (legend top right), and scaled in size to represent task duration (larger diamonds = longer tasks). Diamonds are displayed with a slight vertical “jitter” within each row to reduce overlap between closely timed tasks and enhance visual clarity. This visualization is intended to provide a task-level view of workflow fragmentation and task switching over time, and to illustrate variability in workflow sequencing across observed shifts. *Shifts (n=20) are sorted by total number of tasks (ascending) with observer’s 45-minute break omitted. †Diamond sizes are proportional to duration. Vertical jitter applied to overlapping diamonds for enhanced visual clarity.

## Abbreviations

AI: artificial intelligence
ED: emergency department
EHR: electronic health record
EKG: electrocardiogram
IRB: institutional review board
PA: physician assistant
