# Supplementary figures and images for: Quantifying and Visualizing Emergency Physician Workflow: Observational Time-Motion Study
Source: JMIR Med Inform. 2026 Jul 22;14:e85983. doi: 10.2196/85983 (PMC13397913; doi:10.2196/85983)

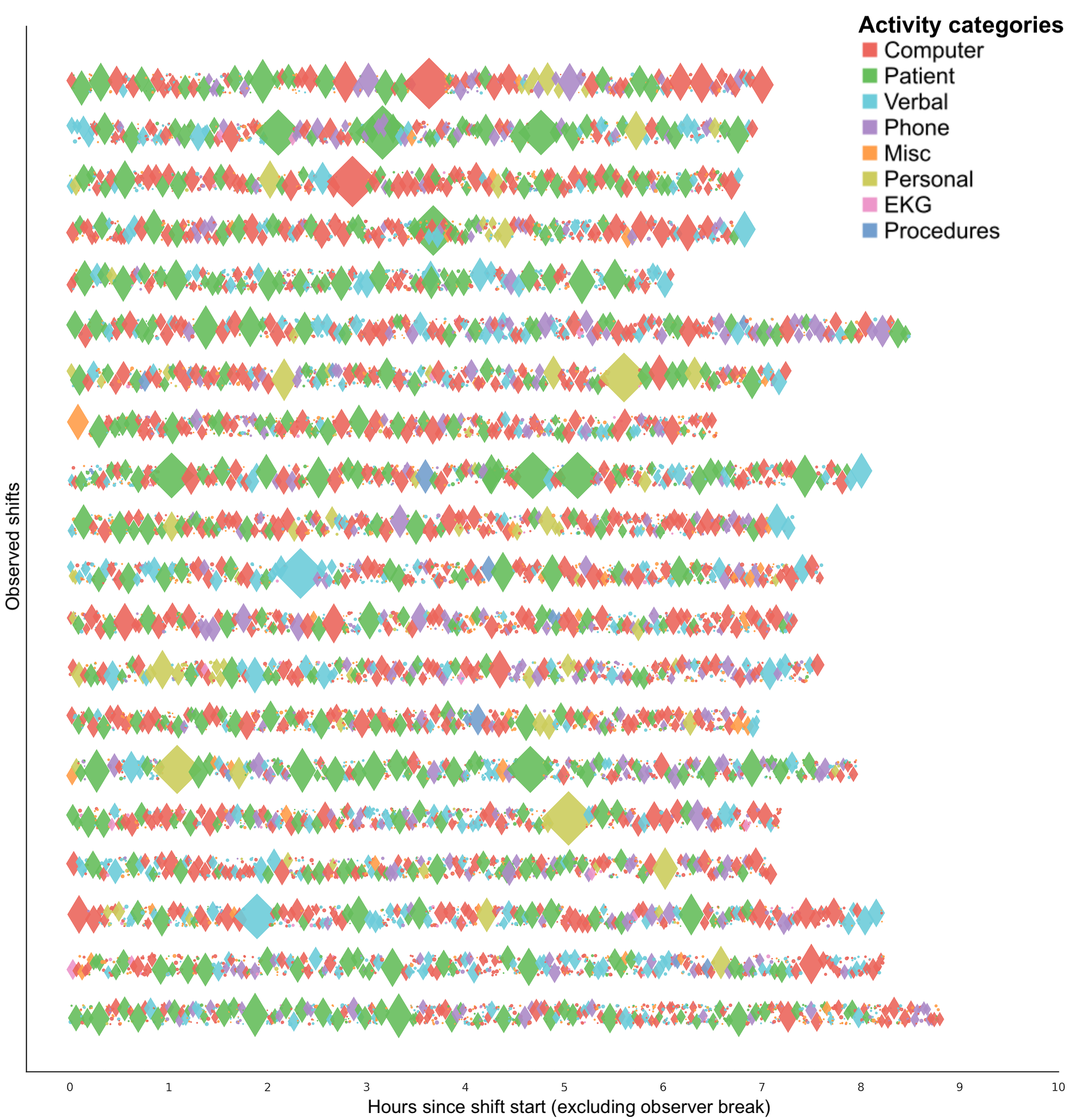

Supplement: Multimedia Appendix 1 [file medinform-v14-e85983-s001.png]
